# Microbial fuel cell compared to a chemostat^☆^

**DOI:** 10.1016/j.chemosphere.2022.133967

**Published:** 2022-06

**Authors:** John Greenman, Buddhi Arjuna Mendis, Iwona Gajda, Ioannis A. Ieropoulos

**Affiliations:** a Bristol BioEnergy Centre, Bristol Robotics Laboratory, University of the West of England, BS16 1QY, UK; b Biological, Biomedical and Analytical Sciences, University of the West of England, BS16 1QY, UK

**Keywords:** MFC, Chemostat, Steady state, Dilution rate, Growth rate, Electrical power

## Abstract

Microbial Fuel Cells (MFCs) represent a green and sustainable energy conversion system that integrate bacterial biofilms within an electrochemical two-electrode set-up to produce electricity from organic waste. In this review, we focus on a novel exploratory model, regarding “thin” biofilms forming on highly perfusable (non-diffusible) anodes in small-scale, continuous flow MFCs due to the unique properties of the electroactive biofilm. We discuss how this type of MFC can behave as a chemostat in fulfilling common properties including steady state growth and multiple steady states within the limit of biological physicochemical conditions imposed by the external environment. With continuous steady state growth, there is also continuous metabolic rate and continuous electrical power production, which like the chemostat can be controlled. The model suggests that in addition to controlling growth rate and power output by changing the external resistive load, it will be possible instead to change the flow rate/dilution rate.

## Highlights

• Novel exploratory model regarding MFC electroactive biofilms akin to a chemostat.
• Presenting “thin” biofilms forming on perfusable anodes in continuous flow MFCs.
• MFC can behave as a chemostat including steady state growth.
• Continuous steady state growth and electrical power production can be controlled by flow rate.

## Graphical abstract

**Figure:**
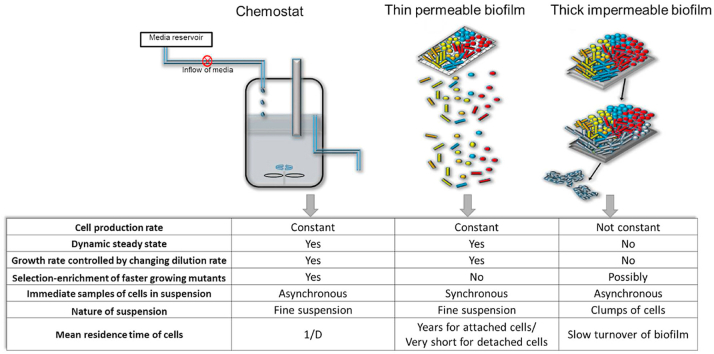

## 1 Introduction

This review briefly covers a description of Microbial Fuel Cells (MFC) focussing on one special type of MFC, assuming thin biofilms (e.g. monolayer) and fed by advective transport of nutrients. This is so biofilms never become limited by the speed of molecular diffusion travelling through multilayers of cells and extracellular polysaccharide (EPS) typically found in a conventional thick biofilm, whose location is somewhere between the nutrient source and the electrode surface. Thin monoculture biofilms (e.g. *Shewanella*) appear to reach a very stable condition growing continuously with constant electrical output for constant physicochemical conditions and at a growth rate proportional to the flow rate. A shift in conditions to a new but constant set of parameters puts the microbes through a period of transition as cells adapt and adjust from one condition to the next, but once adjusted become stable again giving a different constant output for constant conditions. By ensuring that sterile medium is used at the input, detection of cells at the output can be quantified as viable counts, biomass or optical density. This allows the operator to measure the MFC production rate of cells in correlation with power output. We describe a perfusable anode model for cell growth within an MFC, which in theory - and possibly in practice - can be imagined to be a perfect, multi-steady state system that is controlled by the speed of rate-limiting-nutrient flow, set by the investigator. The model assumes great similarity to the chemostat but with important differences, which will be elaborated upon later. We also propose a way to test, validate or measure the steady state from a biofilm, by analysing *synchronicity*. In a continuous flow, thin film MFC, a strong relationship between growth rate, metabolic rate and power output can be assumed, over a wide range of physicochemical parameters; this is especially true when the carbon/energy supply rate (fuel supply) is the main limiting growth factor. By controlling the flow rate, we can control the growth rate in a similar manner to that of a chemostat.

## 2 Chemostats

**Fig. 1:**
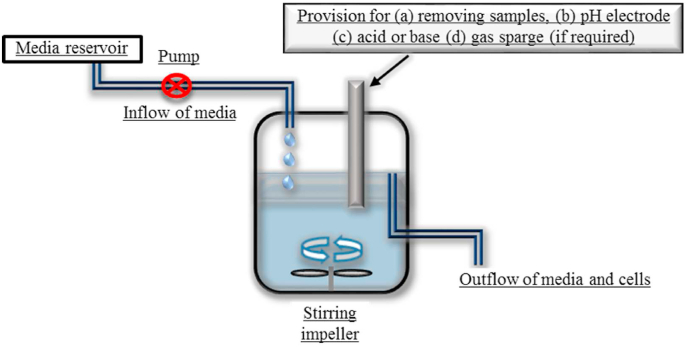
Diagram of the main features of a chemostat.

In 1949, Jacques Monod remarked: “*The study of the growth of bacterial cultures does not constitute a specialized subject or branch of research: it is the basic method of Microbiology*” (Monod, 1949). The chemostat was independently invented by Novick and Szilard (1950) in the US and Monod (1950) in France. Monod used the chemostat as a method of obtaining stability, consistency and reproducibility of culturing microorganisms, whilst Novick and Szilard used the invention to study genetic changes and mutations, focussing more on the chemostat as a way of selecting or enriching for the fastest growing species or strains. The advantages of chemostats over batch culture systems lie in the way that growth can be controlled. In batch culture there are always changes in the physicochemical environment as growth proceeds. The concentration of substrate diminishes whilst the products of metabolism accumulate. The cell number increases exponentially, and the growth rate initially accelerates, stays constant for a time and then decelerates. The changes are arbitrary, depicted by using terms such as lag, exponential, stationary and decline phase. Unless the system is well buffered, there is often a shift in pH and, for aerobic or facultative species, there is a reduction in dissolved oxygen. In empirical scientific research and modelling, the independent variable is the single factor that is manipulated, the hypothesis being that this variable causes a direct effect on observable features called the dependent variables. The idea is to vary the independent variable and watch what happens to one or more dependent variables. This is impossible to achieve using batch cultures, since there are intrinsically too many interactions between all the variables (i.e. conflation of variables). In contrast, for an open flow system, there is a high degree of extrinsic control over the physicochemical conditions by the operator. In a chemostat, the influx of sterile medium from a reservoir is balanced by the efflux of spent medium, living cells and cell debris from the vessel, allowing growth to occur at an equilibrium, with growth of new cells being balanced by those washed out. There is no accumulation or build-up of metabolic products. The degree of control over the physicochemical parameters in a chemostat allows simplification of the growth conditions, allowing just one variable to be manipulated at a time (Fig. 1). This has contributed highly to some important advances in our understanding of elementary microbial processes (Pirt, 1975).

In a modern chemostat, the behaviour of cultures can be accurately monitored using real time measuring instruments and advanced sensor technology. These devices coupled with the use of molecular tools (fluorescent proteins, bioluminescence, isogenic mutants and auxotrophic species), has brought about a revival in the use of the chemostat owing to the advantages presented in environmental control (steady states), reproducibility, and modelling, making it a powerful tool for the microbial physiologist. The most critical feature of a chemostat is that microorganisms can be grown in a physiological steady state under constant environmental conditions. In this steady state, growth occurs at a constant specific growth rate and all culture parameters (volume, nutrient/product concentrations, pH, cell density, temperature, dissolved oxygen (DO), redox remain constant.

Most of the published literature using chemostats has involved the study of pure monocultures. Nevertheless, chemostats can be used to study mixed species communities both ecologically (Pavlou and Kevrekidis, 1992; Becks et al., 2005) where mutation/selection can add further complexity and evolutionary biology (Wick et al., 2002) where mutant selection itself is under study. The rapid development of molecular biology over the last 30–40 years resulted in a decline in the use of the chemostat as a fundamental tool in microbiology although global (post genomic) use for studying microbial processes has now led to a resurgence for studying growth, metabolic pathways, nutrient limitations, and stress responses at the whole-organism level. Biofilm formation within a chemostat is usually considered problematic since biofilm wall growth takes away nutrients and adds metabolic products upsetting the steady state conditions of the planktonic cells. The cell population numbers growing within the biofilm are difficult to enumerate *in situ*. Uptake of the growth rate limiting nutrient by the biofilm population is also difficult to measure and for modelling purposes (Legner et al., 2019; Pilyugin and Waltman, 1999) it is assumed to be the same as the planktonic cells. For thick diffusion-limiting biofilms it may not be so.

## 3 Biofilms

**Fig. 2:**
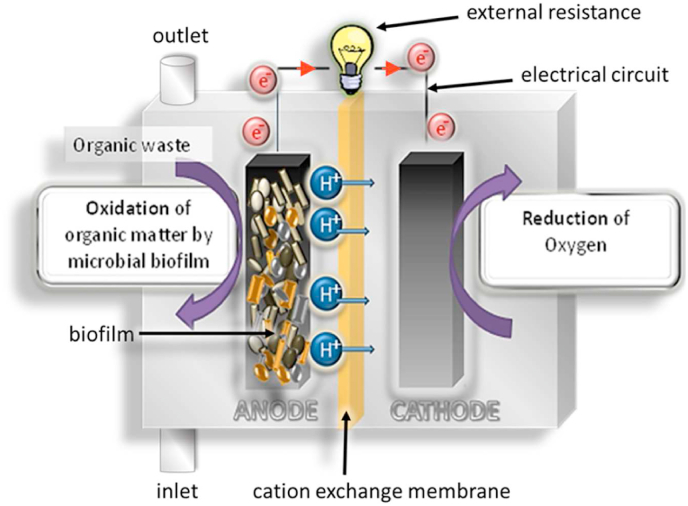
Diagram of a Microbial Fuel Cell with microbial anode operated under continuous supply of organic substrate.

Over 170 years ago it was first observed by Leuwenhoek that microorganisms such as bacteria grow preferentially on surfaces. Since then, the growing research in the area established that bacterial cells can sense their proximity to a surface and actively adhere to the surface to form a biofilm. The biofilm when initially formed contains multicellular microcolonies made of a matrix of communities of one or of many species. As it grows, it develops an assembly of microbial cells associated with a surface and enclosed in a matrix of primarily polysaccharide material as extracellular polymeric substance (EPS) forming a defined architecture (Costerton et al., 1995). The biofilms and their functionality is of importance in both medical (such as antimicrobial resistance) as well as environmental research. Biofilms are key components of an ecosystem functioning actively participate in decomposition of organic matter and nutrient cycling. In natural or artificial habitats, biofilm formation is a strategy protecting microorganisms from environmental hazards. In the emerging field of bioelectrochemistry, biofilm development directly onto the electrode surface (the anode) is the core of the energy transformation where the bacteria convert organic substrates into electrical current. The capability of electroactive microorganisms to donate the electrons originating from their metabolisms directly to the electrode can be illustrated in the Microbial Fuel Cell (MFC) technology that is capable of turning organic matter into electrical energy (Fig. 2).

### 3.1 Electroactive biofilms in MFCs

MFCs are promising technology for the generation of green electricity by converting chemical energy bound in organic matter into direct electrical current thanks to the activity of electroactive bacteria forming a biofilm on the surface of the anode electrode (Fig. 2). MFCs consist of two chambers: an anode and a cathode, separated by a separator or electrolyte. In the anodic half-cell, organic compounds are oxidised by microbial biofilm resulting in production of electrons, protons and CO_2_. Electrons travel via an external electrical circuit to the cathode, while the protons and other cations migrate across a separator to maintain charge balance and combine with electrons on the cathode reducing oxygen. This gives an opportunity for researchers to study the role of biofilm on the anode surface as a function of bioelectricity for the development of biopower sources and biosensors (Santoro et al., 2017).

### 3.2 Detachment of electroactive and other biofilm species

**Fig. 3:**
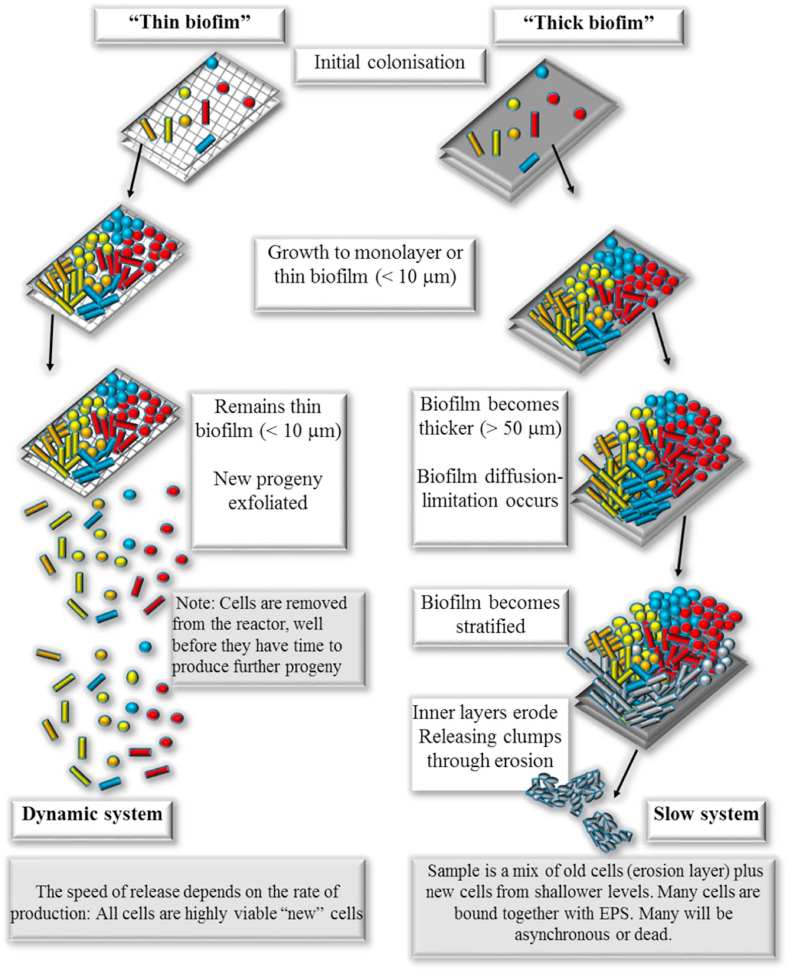
Comparison of “thin” biofilm on perfusible substratum (e.g. carbon veil) and “thick” MFC biofilm on non-perfusible biofilm (e.g on solid graphite sheet).

It is clear that the biofilms that form on a permeable (perfusible) substratum (e.g. carbon veil electrode) and an impermeable substratum (e.g. graphite block) have important differences, particularly with regard to the supply and distribution of nutrients to cells and the nature of the biofilm matrix. At the macro/meso scale, on the impermeable surface, the biofilm matrix that forms becomes thick and stratified, therefore does not allow substrate to reach all layers; in the perfusible system at the macro/meso scale it does due to porous nature of the electrode (Fig. 3). In continuous flow the perfusible system allows nutrients to reach all cells at the same time by advective transport with little in the way of nutrient limitation by diffusion. This is in contrast with a mature biofilm produced on a solid surface that produces a much thicker biofilm (Fig. 3). But even under non ideal conditions (a batch culture system with solid graphite electrodes within a large 225 ml anodic volume), Bond and Lovley (2003) commented on the early stages of colonisation by *Geobacter sulfurreducens*, stating that the “SEM of electrode surfaces recovered at this stage revealed nearly full coverage of the electrode surface by a layer of cells, which was rarely more than a few cells thick”. A study by Read et al. (2010) showed that biofilms of pure culture Gram-ve and Gram + ve species remain viable nearest to the working electrode whilst losing viability on top or further away from the electrode (i.e. during closed circuit operation with flowing current). This was in contrast to when the anode was in open circuit where viability was highest on top of the biofilm, furthest away from the anode. It should be pointed out that in open circuit all electroactive mechanisms stop and the electrode ceases to be the end terminal electron acceptor for the microorganisms. A study by Sun et al. (2016) again, under non ideal conditions of re-cycled batch culture MFC showed that *Geobacter sulfurreducens* biofilm reached the highest electrochemical activity with a biofilm thickness of ∼20 μm. Furthermore, the electrochemical activity decreased with increasing thickness, until the biofilm growth ceased at a thickness of ∼45 μm. Electrochemical analysis and the metabolic spatial variability showed, that in the first 5 cycles the live cells grew fast, which led to a rapid drop of charge transfer resistance and further contributed to high current generation. However, from cycle 5 to 12, a great many inactive cells accumulated in the inner layer of biofilm, resulting in high diffusion resistance, suggesting that the live-cell mass contacting the electrode rather than the biofilm total thickness was responsible for the high current generation. Mclean et al. (2010) used an optically accessible, dual anode, continuous flow MFC to enable real-time microscopic imaging of anode populations of *Shewanella oneidensis* strain MR-1 as they developed on solid electrodes. When a low ohmic value (100Ω) external load resistance was used, estimates of current per cell reached a maximum of 204 fA/cell (1.3 × 10^6^ e^−^ cell^−1^sec^−1^), but only 75 fA/cell (0.4 × 10^6^ e^−^ cell^−1^sec^−1^) when using a higher ohmic resistance (1 MΩ). The 1 MΩ anode biomass consistently developed into a mature thick biofilm with tower morphology (>50 μm thick), whereas only a thin biofilm (<5 μm thick) was observed using the 100Ω load. In essence, the higher ohmic values open circuits the MFC whilst a tuned lower ohmic value applies an adequate resistance forcing the MFC to produce power. Xiao et al. (2017) compared *Shewanella oneidensis* MR-1 cells containing extracellular polymeric substances (EPS) with cells treated to remove EPS and noted that the maximum current increments from EPS-depleted MR-1 were 40–90% higher than that of the control group showing that MR-1 in the absence of EPS can transport electrons more efficiently than in the presence of EPS. More recently, the same phenomena (high external resistance load, thick biofilm and low power output vs low resistance load, thin biofilm and higher power output) was observed by Pasternak et al. (2018), but using more diverse, mixed culture MFCs.

Experiments using small scale MFCs with a perfusable carbon veil electrode (anode) and increasing or decreasing the flow rate of feedstock medium in steady state stages, demonstrated how the supply rate of carbon energy limited medium can “control” the microbial growth rate. Dilution rates as low as D = <0.01 h^−1^ resulted in very low power, whereas high flow, with D up to 1 h^−1^ resulted in higher (but not highest) power output. Highest power was recorded at a growth rate of 0.85 h^−1^, which is assumed to be the maximum specific growth rate (or μ_max_ value) for the monoculture species *Shewanella oneidensis* (Ledezma et al., 2012). The value for μ_max_ (0.85 h^−1^), is higher than the assumed or suspected growth rates occurring in most thick biofilm systems. The finding supports the view that the fastest growth rate relates to the highest metabolic rate and therefore greater rates of all biotransformation. The search for better performance in terms of electricity generation is the easiest way to search for best performing species for recycling all the elements taken up by living cells. Carbon energy (C/E) is respired at the electrode to create energy and take up mineral elements such as: [N, P, K, S, Mg, Fe, Mn] and speed up anabolism, where these minerals are recycled into new biomass. High power by the cell is not antagonistic to highest levels of recycling. There is no competition between the electrical energy output on the one hand and rates of uptake or “treatment” of organic material on the other. If the fastest growth rate relates to the highest metabolic rate and greater rates of all biotransformation then it would be resulting in better performance within an MFC therefore the MFC output can be used as a metric of the metabolic rate and a selection tool that will enable the growth of the fastest growing organism.

The question of whether diverse mixed species can perform in a similar manner to a pure culture model then the answer is they can still outperform less diverse flora in their ability to tackle a wide range of substrates including polymers. It is known that the performance of constantly flowing MFCs have been monitored at least in one experiment for over 13 years by current authors (Ieropoulos et al. *unpublished data*) and show good long-term stability of output levels. However, constancy of output between steady states does not necessarily mean that the electrode is ecologically stable through many states and their transitions. It is known that many species can compete with many others in colonising a new ecological niche. Experiments using defined mixed cultures (e.g. *Shewanella* plus *E. coli*) will show promise for extending the spectrum or range of potentially utilisable C/E substrates. *Shewanella* can feed off the fermentation products from *E. coli* that can hydrolyse polymers that *Shewanella* alone cannot utilise otherwise (Yang et al., 2015). Small molecules like acetate and lactate are the main end products of many fermenting species, including *E. coli*. Some heterotrophic anaerobes can digest cellulose and it is important to note that without such a species, cellulose cannot be utilised.

## 4 Theories and models

A number of theoretical models have been proposed to account for the phenomena occurring in biofilms, including the biofilms used in MFCs. The advantages of biofilms forming on an electrode means that any electroactive properties of the microbes can be used to measure or monitor its progression through time. The higher the rates of production the higher the reducing power of the cell. The electrical power output corresponds to the microbes’ reducing power, ultimately provided by metabolism of NADH, NADPH, and other redox chemicals (FADH, glutathione, cysteine) within the growing cell, but the totality is simply called “cell reducing power” (Hastings et al., 1922; Russell, 2007). The unit of measurement is Watts (W or mW or μW or pW) or for a continuous system in W×hours; likewise, the MFC electrical power output, measured from the MFC circuit is expressed in W (or mW or μW or pW)). Cell reducing power and MFC electrical output are inextricably linked. The only exceptions would be if the cells were not being grown under C/E limited conditions. An example might be when C/E is added at high concentrations, when the cells are replete with reduced organic substrates and are limited instead by a shortage of one or more of the essential elemental nutrients such as nitrogen, phosphate, sulphur, potassium, or magnesium. Under these conditions (excess carbon) anabolism and catabolism in the cell becomes uncoupled (Russell, 2007) and the cells become limited by the rate of re-oxidation of NAD(P)H back into their oxidised form NAD(P)^+^. Carbon excess switches the cell to produce storage chemicals, particularly cell wall capsular polymers or slimes. Some species store granules of glycogen (Moradali and Rehm, 2020). If phosphate is in excess, many species will store polyphosphate or other polymers (Moradali and Rehm, 2020). These may well change the model from being a thin biofilm to forming a thick biofilm with increasing diffusion limitation and consequent slowing of growth rate. It is likely that anodophiles on electrodes would simply get rid of excess reducing power by producing higher electrode output, but this remains to be determined.

The concept of maintenance and its impact on growth rate and yield became even more complicated when nutrients other than C/E (glucose) were used to limit growth (Teixeira de Mattos and Tempest, 1983; Streekstra et al., 1987). If continuous cultures were nitrogen-, sulfate-, potassium or phosphate-limited, the ‘apparent’ maintenance value was 10-fold higher. With regard to heterotrophic species it appears that it takes less energy to maintain a glucose limited cell than it does for a cell grown in glucose excess conditions. By the 1980s, it became apparent that many bacteria used another avenue of energy dissipation (energy spilling) that was distinct from maintenance (Russell and Cook, 1995).

Growth studies using *Streptococcus bovis* indicated that energy spilling was not just a characteristic of resting cells. Glucose-limited continuous cultures did not spill energy, but with glucose excess conditions rapidly growing cells spilled as much as 25% of their ATP (Russell, 1991). In bacteria and other living cells, the ratio of ATP to ADP changes little until the cells are starved and dying. *E. coli* uses cAMP as a signal to regulate the transcription of certain proteins (most notably lactose permease). Moreover, the metabolic end product of energy spilling itself can be a valuable product. Such is the case in ethanol and solvent production. In both cases, large amounts of product are not produced until growth has ceased (Cot et al., 2007; Russell and Diez-Gonzalez, 1998).

It is unknown to what extent energy spilling and the cells high requirement for maintenance energy is affected when anabolism is not tightly coupled with catabolism. Although not tested empirically in MFCs, it seems that C/E-limited conditions will allow for higher values for growth rate and substrate utilisation efficiency, with less chance of inefficiency due to unbalanced requirements during a period when anabolism is not strongly coupled with catabolism.

### 4.1 Chemostat models

In a chemostat, different models have been proposed to describe the growth kinetics of cells growing in steady state when limited by a single growth limiting nutrient (Wade et al., 2016). The Monod model is favoured since the parameters involved, specific growth rate μ , maximum specific growth rate $\mu_{max}$ of bacteria, the rate limiting substrate concentration S, the substrate affinity constant $K_{s}$ have real world counterparts whose meaning and empirical measurement are experimentally accessible.

Considering a simple system such as the chemostat, with a total biomass X, and volume V, fed with a constant volumetric flow rate of f with an initial substrate concentration of $S_{0}$,

then $D=\frac{f}{V}$ is defined as the dilution rate, in which the specific growth rate is a function of time dependent concentration S(t). Assuming $DS_{0}$ is the input rate that nutrients are added to the container, and then a yield parametery is proportional to the total (net) yield Y;y∝Y; $Y=\frac{\text{mass of bacteria change}}{\text{mass of consumed nutrient change}}$

Based on the above, mass balance equations can be written as follows,

$$\begin{array}{l} \dot{X}\left(t\right)=\mu (S\left(t\right))X\left(t\right)-DX\left(t\right)\\ \dot{S}\left(t\right)=DS_{0}-DS\left(t\right)-\frac{1}{y}\mu (S\left(t\right))X\left(t\right) \end{array}$$

The Monod equation is an empirical manifestation of the general form of Michaelis–Menten equation, for the modelling of microbial growth with Monod constant $K_{m}$ in place of Michaelis constant$K_{mm}$. Monod constant $K_{m}$ is defined as the concentration of substrate, that allows cells to grow at $1/2\mu_{max},$ this is synonymous with affinity constant $K_{s}.$

(1) $$\mu \left(S\right)=\frac{\mu_{\max}S}{K_{m}+S}$$

In a chemostat at steady state, the time dependent mass of bacteria $X\left(t\right)=\bar{X}$ and time dependent concentration $S\left(t\right)=\bar{S}$ will be constant for which $\dot{X}\left(t\right)=0$ and. $\dot{S}\left(t\right)=0$

Thus, specific growth rate becomes the dilution rate.

(2) $$\mu \left(\bar{S}\right)=D$$

Also, $\mu \left(S\right)=\frac{\mu_{\max}S}{K_{m}+S}$ becomes dilution rate and at steady state the rate of substrate utilisation $r_{ut}$

(3) $$D=\frac{\mu_{\max}S}{K_{m}+S}$$

is defined as

(4) $$r_{ut}=-\frac{{r_{ut}}_{max}.S}{K_{m}+S}X\left(t\right).$$

with $\mu_{max}={r_{ut}}_{max}Y,$ where ${r_{ut}}_{max}$ is the maximum specific rate of substrate utilisation.

Maintenance energy:

Theoretical wash-in and wash-out curves are important when switching feedstocks. Washout curves can be used to determine $\mu_{max}$. The fluidic behaviour of an ideal flow-through system is described by the theoretical wash-out (5) and wash-in (6) curves, calculated by the following equations (Pirt, 1975):

(5) $$S=S_{0}e^{-Dt}$$

(6) $$S=S_{0}\left(1-e^{Dt}\right)$$

For continuous culture system of constant volume, the following balance equations can be formulated to determine the concentration of biomass (x). The limiting balance equations can be formulated for biomass and the limiting substrate concentrations as: x=biomass/volumex=X/V. Steady state mass balance equations gives: $-fx+r_{x}V=0$ at steady state, where the cell growth kinetics $r_{x}$ is defined as $r_{x}=\mu x$ (assuming cell growth rate is higher than cell death rate).

$$fS_{0}-f\bar{S}-\frac{1}{Y_{x/s_{c}}}\mu xV\;with\;\mu =f/V=D$$

(7) $$x=Y_{x/S_{\max}}\left(S_{0}-\bar{S}\right)\;DS_{0}-D\bar{S}=\frac{Dx}{Y_{x/S_{\max}}}\text{ with }\mu =D$$

By substituting x from (7) to Monod's equation,

(8) $$S=Y_{x/S_{max}}\left(S_{0}-\frac{K_{m}D}{\mu_{max}-D}\right)$$

If $D>\mu_{max}$ the denominator $\left(\mu_{max}-D\right)=>0$ then, the maximum dilution rate can be found at x=0,

(9) $$D_{max}=\frac{\mu_{max}S_{0}}{K_{s}+S_{0}}$$

In practical systems, $S_{0}$ is significantly greater than $K_{m}$; then the optimal biomass production occurs at $D_{optimal}\approx \mu_{max}\approx D_{max}$ ; which is near washout conditions.

It must be noted that in the above presented analysis the organism is assumed to adapt to the new dilution rate instantaneously and begin to grow at its maximum rate. This assumption is only reasonable for small steps in D (Pirt, 1975). For big step size, the organism needs a considerable adaptation time to reach balanced growth and exert maximum growth rate. During such an adaptation period, the cells will wash out faster than expected, resulting in the calculation of a $\mu_{max}$ value smaller than actual. A small step in D, on the other hand, might result in a prolonged state of substrate limitation or negate the assumption of $\;S\gg K_{m}$, for a considerable period of time following the setup. To avoid this complication, Tempest (1970) recommends the use of a large step increase in D to a culture which has been kept at steady state very close to the true $\mu_{max}$. Therefore, the experimental conditions must be chosen with care. For calculating $\mu_{max}$ in washout phase, a graph of the washout of cells is constructed and $\mu_{max}$ is calculated from the slope of the linear decline in the natural log of cell concentration with time, since maximum specific growth rate can be calculated as:

$\mu_{max}=D+$*slope of the washout curve* {Note that the slope of the washout curve is measured as a negative slope, so the value is actually subtracted from D}.

### 4.2 Biofilm models

Mathematical models that regard biofilms as homogeneous steady state biofilms containing a single species have been proposed (Rittmann and McCarty, 2001). This model has been evolved to cover dynamic multisubstrate and multispecies biofilm computer models (Rittmann and Manem, 1992; Wanner and Gujer, 1986; Wanner and Reichert, 1996). An approach using discrete cellular automata (to simulate the rules that govern the lives or “properties” of microbial cells) has also been employed to model biofilms (Picioreanu et al., 1998; Wimpenny and Colasanti, 1997). These allow the simulated biofilm structure to evolve as a self-organisation process emulating how real bacterial cells organise themselves into biofilms. These models produce realistic, structurally heterogeneous biofilms. Mathematical models of diffusion/reaction in biofilms (incorporating Fick's law of diffusion) have also been described (Dibdin et al., 1996; McNee et al., 1982) as have models to predict the effects of antimicrobial activity within biofilms (Dibdin et al., 1996; Stewart, 1996).

### 4.3 Biofilm based bio-reactors

Biofilms are layer like aggregations of microbes with their extracellular polymers attached to solid surfaces; biofilms are naturally immobilised cells. Using Monod's equation, the rate of substrate utilisation $r_{ut}$ on a chemostat with suspended microbial culture can be defined as in equation (4). The substrate utilisation for a biofilm takes the same form. For a biofilm with bulk liquid concentration S, the substate utilisation is given as:

(10) $$r_{ut}=-\frac{{r_{ut}}_{max}S_{f}}{K_{m}+S_{f}}X_{f}\left(t\right),\;\text{in which}\;S_{f}<S$$

where $X_{f}$ is the total active biomass ***within the biofilm*** and $S_{f}$ is the substrate concentration at the point of the biofilm.

If $S_{f}>0$ at all points of the biofilm, then the biofilm is assumed to be *shallow*. If the shallow biofilm has negligible gradient, then the biofilm is called *fully penetrated* in which the concentration of outer surface $S_{os}$ and of attached surface $S_{as}$ are near identical $S_{os}\approx S_{as}$.

The mass balance equations for substrate in a biofilm under steady state conditions. is given by:

(11) $$D_{mdo}\frac{d^{2}S_{f}}{dz^{2}}-{r_{s}}_{max}.\frac{X_{f}S_{f}}{K_{m}+S_{f}}=0,\;\text{with boundary conditions }{\frac{dS_{f}}{dz}|}_{z=L_{f}}$$

where $D_{mdo}$ is the molecular diffusivity of substrate into the biofilm (Rittmann and McCarty, 2001)

The mass balance equations for substrate transport to biofilm are given by:

(12) $$J=\frac{D_{mdo}}{L_{f}}\left(S-S_{f}\right)$$

Where substrate flux J into the biofilm is the (sum) of reaction rates per unit surface.

For a steady state, $X_{f}S_{f}$ remains constant over time. A steady state biofilm can be defined by the mass balance of

(13) $$YJ=b^{\prime }X_{f}L_{f}$$

where YJ defines the growth rate per unit surface area, the analogous parameter to growth rate in a suspended media, $X_{f}L_{f}$ is the biomass per unit area, $b^{\prime }$ is the overall biofilm specific loss rate, and $L_{f}$ is the thickness of the biofilm – the boundary conditions are defined in relation with the thickness. $X_{f}L_{f}$ and $L_{f}$ can be found by rearranging (13), where biofilm losses $b^{\prime }$, represent the maintenance energy b (which is the decay) and biofilm detachment$b_{det}.$

(14) $$b^{\prime }=b+b_{det}$$

The model for the steady state biofilm requires simultaneous solution of mass balance equations for substrate in biofilm (11), mass balance equations for substrate transport to biofilm (12) and (13) for active biomass in biofilm. The system is solved using a pseudo analytical method as described in Rittmann et al., (Rittmann and McCarty, 2001). Analogues to a steady state suspended microbial reactor such as continuous stirred tank reactors (CSTR), a steady state biofilm reaction can be analysed in a complete mixed biofilm reactor (CMBR).

Assuming a CMBR active reactor with volume V, influent volumetric flow rate $f_{i}$ carried substrate with initial concentration $S_{0}$, an active biofilm surface of an area A, local uniform biofilm thickness $L_{f}$, effective diffusion layer thickness $L_{diff}$, Biofilm accumulation per unit surface area: $X_{f}L_{f}$, effluent flow $f_{e}$, $J_{s}$ the substrate flux and effluent and liquid volume has the same concentration S, active biomass concentration $x_{a}$ (viable within the biofilm) in the reactor and the effluent is at concentration made out of the cell detachment from the biofilm and all microbial activity are assumed to be active in the biofilm.

For a CMBR with a steady state biofilm, substrate utilisation can be determined by:

(15) $$r_{ut}=-\frac{{r_{ut}}_{max}S_{f}}{K_{m}+S_{f}}X_{f}\left(t\right)\;with\;\mu_{max}={r_{s}}_{max}Y$$

Assuming a steady state biofilm where biomass per unit surface area $X_{f}L_{f}$ is constant,

the effluent and liquid volume concentration is given by $S=S_{0}-\frac{J_{s}A}{f_{i}}$ and biomass per unit area as $X_{f}L_{f}=\frac{YJ}{b^{\prime }}$, whereas biofilm thickness can be given by $L_{f}=\frac{YJ}{X_{f}b^{\prime }}$ and active biomass concentration by $x_{a}=\frac{b_{det}X_{f}L_{f}V}{f_{i}}$. The substrate concentration $S_{f}$ of profile of a biofilm is nonlinear. In a deep or thick biofilm the substrate concentration becomes zero at some point in the film nearer to the attached surface, after which the substrate is not utilised, thus further increasing the biofilm thickness would not increase the overall substrate utilisation.

### 4.4 MFC anodic biofilms on perfusible electrodes

Bio Energy reactions for MFCs:

Microbial cells obtain their energy for growth and maintenance from redox reactions. Redox reactions involve an electron donor (reduced organic substrates) and an acceptor. The common electron acceptor under aerobic conditions is molecular oxygen, but under anaerobic conditions, the anode electrode is able to act as the electron acceptor.

Although there have been published a large number of theoretical models for MFC (Xia et al., 2018), most have focussed on thick conventional biofilms and few have considered the continuous flow matrix perfusion electrode free of diffusion limitation. In these models, following transfer of inoculum, cells attach themselves to the substratum (the matrix) and sterile perfusate supplies the cells by advective transport with nutrients and buffering set or controlled by the researcher. The matrix population begins to grow until a matrix limit (a population saturation point) is reached. This may be more or less equal to the yield of cells, expected from the concentration of rate limiting substrate but it is more likely that it is governed by the occupancy limit that is the numbers of attachment sites that are available, and once attached will remain constant rather than increasing.

If it is assumed that the perfusion matrix allows flowing medium to reach all cells at the same time, there should be no delay of transport and should be no decay of the biofilm. Biofilm modelling of deep biofilms includes both synthesis of new biomass, and decay. If it is assumed that the biofilm is a monolayer or shallow biofilm, then it does not decay; there is no diffusion limitation, then only the synthesis part of bacterial dynamics is considered, and the decay rate is instead designated as the rate of detachment of new progeny from the biofilm.

Due to the difficulty of analytical solutions and the complexity of the biofilm models, such as the MFC biofilm model, which is akin to the biofilm model of a CMBR in the case of a shallow (thin) or fully penetrated biofilm; the simpler chemostat equations which are ideally meant for a suspended biomass, are used with the assumptions that they are suited for a perfused electrode, in order to simplify the process of analysis in terms of biomass generation and growth rate. This has been demonstrated theoretically by Torres et al. (2010) and experimentally by previous research (Cai et al., 2018; Rittmann and McCarty, 2001).

Assuming that the rate of generation of the biofilm is equated to the growth rate defined by Monod. (1) The growth rate can be (*written as or renamed as*) the rate of synthesis ($\mu_{syn}$) and can be written as:

(16) $$\mu_{syn}=\frac{1}{x_{a}}\frac{dx_{a}}{dt}=\frac{\mu_{max}S}{K_{m}+S}$$

In a diffusion limiting biofilm, $x_{a}$ is the expression for the active biomass concentration. Since the freshly detached daughter cells in our model are devoid of an end terminal electron acceptor, they will not continue to divide but will be washed away. The biofilm cells are assumed to be 100% viable, so $x_{a}$ can be written as $x_{bio}$ (the attached biomass).

The Monod equation can also be used to derive substrate utilisation $r_{ut},$ when it takes the form of:

(17) $$r_{ut}=-\frac{{r_{ut}}_{max}S}{K_{m}+S}x_{bio}$$

Describing mass balances and the rate of substrate concentration change requires specifying a control volume. The liquid volume of the MFC is denoted as V. The system receives a feed flow with rate f, having an initial substrate concentration of $S_{0}$, which is described as the MFC initial substrate concentration.

The rate of change in substrate concentration in the MFC is,

(18) $$\frac{dS}{dt}=-r_{utmax}+D\left(S-S_{s}\right)\;with\;D=\frac{f}{V}$$

The dilution rate D, is the control input to the system. By changing the flow rate, one can examine the effect of D on the cell and power outputs of the MFC since the dilution rate is a function of the flow rate. In the continuous system, the biomass mass balance equation is:

(19) $$\frac{dx}{dt}=\mu x_{bio}-Dx_{p}$$

where, $x_{p}$ is used for progeny in the planktonic phase. Again, the parameter μ represents the net specific growth rate of the attached bacteria. The relationship between limiting substrate and biomass yield is more complicated, since the model assumes a non-accumulative (i.e. constant population) steady state. All the yield of cells is contained within the output of daughter cells not by the accretion of new biofilm. $\mu =\mu_{syn}=\mu_{p}$ where $\mu_{p}$ is the production rate of new cells from the biofilm shed into the perfusate.

The link from substrate utilisation to power production is given by Torres et al. (2010). For an MFC, the current density (j) is given as:

(20) $$j=j_{max}\frac{S}{K_{m}+S}.$$

where S is the substrate concentration and $K_{m}$ is the Monod's constant and $j_{max}$ is the maximum current density of the anode. This can be expressed in power providing the voltage is known to be constant throughout the range of steady states. At moderate to high flow rates at maximum power transfer point (controlled by the value of the external load resistor), then for power (P) the equation becomes:

(21) $$P=P_{max}\frac{S}{K_{m}+S}$$

### 4.5 MFCs compared with chemostats

Both systems have a controlled volume which contains both the organic substrate matter and the microorganisms. The proposed MFC anode biofilm model considers only one kind of microorganism and one type of substrate (e.g. lactate) but the approach might be amenable to generalisation for a mixture of microorganisms and a mix of types of substrates. The main assumptions are that: 1. The anode electrode is the only end terminal electron acceptor of significance in the chamber (levels of oxygen, sulfate and nitrate are low or non-existent); 2. The mixing of substrate into the biofilm is ideal, and the substrate gradient within a thin perfusible biofilm is neglected; 3. The substrate concentration change from input to output and the supply flow rates are the main parameters affecting the biofilm growth rate and power output; 4. The temperature remains constant, and the pH is kept constant via buffering or pH controller (pH auxostat) or medium composition where cells produce acid and base in equal measure; 5. The main overpotential affecting the cathode potential is the activation loss. For simplification and because of the small changes in the cathode open circuit potential (OCP), the cathode OCP is assumed to be constant; 6. The substrate input is sterile; there is no addition of active biomass to the biofilm. All cells that leave the system are a product of the biofilm. Cells that detach from the anodic biofilm are devoid of an end terminal electron acceptor so become relatively inert and wash out at a rate depending on the flow rate; 7. Although MFC can be compared to a chemostat in that growth rate is proportional to flow rate (rate of substrate supply), there are some important differences. For example, in a chemostat when dilution rate D is set beyond $\mu_{max}$ , there is eventually complete washout of cells from the system. In contrast, for both a CMBR and an MFC with biofilms, at dilution rates beyond $\mu_{max}$ there are still growing cells contributing progeny and so the system does not wash out although there is a dilution effect and a thinning of the biomass released.

### 4.6 Perfect steady state biofilm model

A perfect model assumes that the layer of attached cells are such that once colonised, their binding to the electrode is sufficiently strong that they do not get removed/replaced when fed fuel unless the cells are killed, lysed and hydrolysed; yet they are all fully viable and growing new cells providing they are fed with rate limiting substrate. In a perfusion electrode, all attached cells have access to nutrients and substrate molecules at the same time, which are brought to the cells by advective hydrodynamic flow. It is the supply rate of limiting nutrient rather than biofilm diffusion that controls growth rate, and there is little in the way of diffusion barriers for a thin monolayer (or thin layer) of cells that form around fine fibres of carbon that make up the carbon veil electrode. For a small scale MFC (e.g. anodic volume = 5–10 ml) biofilm, the rate of supply is what dictates growth rate.

If the kinetics of bacterial growth in a chemostat (Herbert et al., 1956; Powell, 1956) are considered, organisms are contained in a culture of fixed volume (V) to which fresh medium is pumped at a constant flow rate (f), the dilution rate (D) being given by the ratio f/V. The net change in concentration of biomass (x) in the culture vessel with time will thus depend on the relative rates of bacterial synthesis (μ) and ***‘washout’*** rate of organisms from the culture vessel which equals to the dilution rate (D); that is.

Change = Growth – Washout i.e. $\frac{dx}{dt}=\mu x-Dx_{wash}$

Assuming at steady state that the washout biomass is equalling to the growth biomass:

(22) $$\frac{1}{x}\frac{dx}{dt}=\mu -D=0$$

When the culture is in a steady state equilibrium condition, the growth rate and dilution rate are equal and so the net change in the concentration of organisms with time is zero. Now, if cell synthesis was made to cease suddenly the concentration of planktonic organisms in the culture would diminish (through washout) at a rate proportional to the dilution rate; that is,

(23) $$\frac{x_{t}}{x_{0}}=e^{-Dt}.$$

where $x_{0}$ is initial biomass concentration and $x_{t}$ is biomass concentration at time t. For chemostats increasing the loading with the change of $S_{0}$, f, or dilution rate, resulting in the decrease of hydraulic retention time eventually results in biomass washout. In a CMBR, the biomass remains attached to a surface, until the upper limit of loading where $S/S_{min}$ approaches $S_{0}/S_{min}$, after which the biofilm remains, the rate of substrate removal becomes insignificant compared to the supply rate $fS_{0}$.

### 4.7 Growth yield

True yield (Y) is defined as an index or proportion that relates the mass of new cells (active) to the amount of substrate utilised:

(24) $$\frac{dX_{a}}{dt}=Y\left(\frac{-dS}{dt}\right)-bX_{a}$$

where the rate of change of $X_{a}$ is the net growth rate of active biomass, negative rate of change of substrate is the reduction of substrate, b is the decay rate. The net yield can be defined as

(25) $$Y_{n}=Y-b\left(\frac{X_{a}}{-dS/dt}\right)$$

With the net yield $Y_{n}<Y$ due to a portion of energy in the substrate being consumed as cell maintenance. If the rate of substrate utilisation $r_{ut}$ is sufficiently low, then the energy is primary utilised for cell maintenance then the net yield becomes zero, with $r_{ut}$ only adequate to maintain the cells, with no net growth:

$\left(\frac{-dS/dt}{X_{a}}\right)=\frac{b}{Y};Y_{n}=0,$ where, the maintenance energy is defined as. $E_{m}=\frac{b}{Y}$

The active cell mass $X_{a}$ meaning biomass is the collective sum of new progeny over a unit time period, divided by the amount of substrate consumed during the same period of time. It is a dimensionless equation providing mass of cells and mass of substrate are measured in the same units. A yield of 10% (Y=0.1) means that only 10% of the substrate is being used to make new cell material, the rest is used for energy production. An expression for substrate utilisation rate can be obtained by incorporating the total yield index Y with Monods eqaution (1),

(26) $$r_{ut}=\frac{-{r_{ut}}_{max}S}{K_{m}+S}X_{a}$$

With defining $\mu ={r_{ut}}_{max}Y$ with ${r_{ut}}_{max}$ as the maximum specific rate of substrate utilisation. This expression can be changed for conditions where substrate is well below $S\ll K_{m}$ (i.e. when substrate is very low) and the equation can be approximated to first order kinetics. $r_{ut}=\frac{\mu X_{a}}{Y}$

### 4.8 Coulombic efficiency

The coulombic efficiency, is defined as the ratio of total Coulombs actually transferred to the anode from the substrate, as a percentage or ratio to the maximum number of Coulombs possible if all substrate produced current. The total Coulombs obtained is determined by integrating the current over time. For a continuous flow system, the equation used by Logan et al. (2006) is favoured:

(27) $$\varepsilon_{CB}=\frac{MI}{Fbq\Delta COD}$$

where $\varepsilon_{CB}$ is the coulombic efficiency, withM = 32, the molecular weight of oxygen, I = current from Ohm's law, F is the Faraday constant, b=4 which is the number of electrons exchanged per mole of oxygen, q is the volumetric influent flow rate, COD is the measure of substrate in terms of chemical oxygen demand and ΔCOD is the difference between the influent and effluent levels, that which is utilised. The coulombic efficiency is diminished by any utilisation of alternate electron acceptors, either by the bacteria present in the medium (or wastewater), or those in the MFC if oxygen is available, e.g. by diffusing through the membrane. Methanogens use $CO_{2}$ as an end terminal acceptor, and some fermentation species are unable to utilise the electrode as an electron acceptor yet will still utilise COD.

### 4.9 Linear/exponential growth model & calculation of biofilm specific growth rate *μ (h*^*−1*^*)*

In steady state, the production rate of cells from the MFC (cells $h^{-1}$) is measured by taking the output flow. The number of counted cells $mL^{-1}$ is then multiplied by the flow rate in $mLh^{-1}$ to give total production rate of cells per hour. This is the production rate of the MFC as a whole unit. To harvest the attached cells from steady state biofilms, the anode electrode substratum matrix (from MFC) is removed as a whole or a representative sample of the whole is removed, briefly dipped or softly rinsed in buffer (to remove any nonadherent cells) but then vigorously vortexed for 5 min in 10 ml of sterile diluent (saline, or PBS, or 0.1% tryptone yeast extract) to remove the adherent cells (Ledezma et al., 2012). For very adherent biofilms the inclusion of small sterile ballotini beads during the vortex mixing stage may improve the extraction rate or one can use sonication, but the latter decreases the viability of the sample. Viable counts and/or microscopic counts are then performed giving the value of the total biofilm population. Vortex mixing for 5 min has been shown to remove 99% of adherent *E. coli* cells from cellulose acetate membrane biofilms (Evans et al., 1990). By measuring the production rate of cells over time both the MFC and/or perfused substratum system can be shown to be at steady state. For the MFC the power output can also be monitored to show steady state since the power output is directly proportion to the metabolic rate ($Q_{met}$) which in turn is directly proportional to the growth rate.

Two methods have been used to obtain the growth rate of the biofilm ($\mu_{bio}$):

1. The method of Gander and Gilbert (1997):

The doubling time ($t_{d}$) of the cells on the perfusion substratum can be calculated from the following equations: $t_{d}$ = biofilm population/rate of elution of cells. The specific growth rate.

($\mu_{bio},h^{-1}$) of the biofilm may then be calculated using the following equation: $\mu_{bio}=\log\left(e^{2/t_{d}}\right)$.

2. The method of Greenman et al. (2005): $\mu_{bio}=\frac{production\;rate\;of\;cells\;\left(h^{-1}\right)}{total\;biofilm\;population}$

**Fig. 4:**
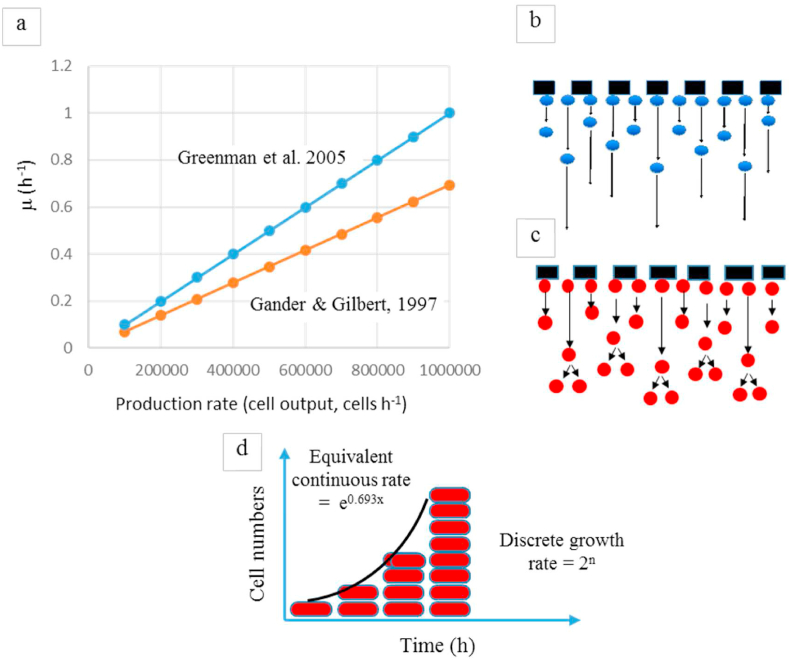
Two approaches to empirical measurement of μ and $\mu_{max}$ using (1)Gander and Gilbert (1997) and (2) Greenman et al. (2005). (a) Shows difference between plots of growth rate (μ) and production rate of cells from the output; (b) shows release of cells but insufficient time for released cells to divide before leaving the reactor (released cells are in synchrony). (c) Shows asynchronous release and further division before leaving the reactor. (d) Illustrates continuous and discrete growth rates.

Using the same data for the biofilm population and rates of production it can be seen that the two methods do not match (see Fig. 4). The first method gives the continuous growth rate whilst the second method expresses the discrete growth rate. For discrete growth, the change in number happens after the specific event of fission. The growth cycle of the cell is completed and the cell divides into 2 cells. With continuous growth, change is *always* happening. There is no single point where change is apparent. Mathematically this is expressed as $2^{x}=e^{ln\left(2x\right)}=e^{0.693x}$. In other words, 100% discrete growth (doubling every period) has the same effect as 69.3% continuous growth. To get to the same production rate of cells from the MFC, continuous growth requires a smaller cell division rate because of compounding due to released cells continuing to divide. The reason for preferring discrete growth is because new daughter cells (being devoid of an end terminal electron acceptor) would be very slow growing in comparison to the attached cells, and at moderate to fast flow rates will rapidly wash out of the small volume reaction vessel at a rate of their production (i.e. biofilm growth rate). The released cells do not have sufficient time to produce further offspring by doubling. This is particularly true if you consider that the time taken to produce a sample (e.g. < 2 min) all the released daughter cells are at the same time point in their individual growth cycle; just after septum formation and separation, so they will all be synchronous (Helmstetter and Cummings, 1963). Within the time period of sampling, the cells are released and will not divide again until their mean doubling time is reached, which is likely to be at least an hour. There is therefore no compounded accumulation within the reactor.

### 4.10 Growth rate in MFC

There is thought to be a direct relationship between population number of active cells on the electrode and electrical power output. This is in line with first order catalytic cell theory that twice the number of cells (or enzyme molecules) will give twice the rate of biotransformation providing substrate is supplied at high rate. At the design stage this can be achieved by increasing the cell binding surface area of the substratum (i.e the electrode) in comparison to the chamber volume, or by growing thicker biofilms. Growing thicker biofilms has its limit because of diffusion limitation whereas increasing electrode surface area has its (eventual) limit due to compaction. But over the course of biofilm development the population number start low and increases with time in fairly strict proportion with the power output. Increasing the area of the surfaces by convolution at the mesoscale (e.g using a mesh of thin wire such as carbon veil) or nano-roughness of each wire on the mesh are also both possible. The latter process increases surface area at the molecular scale favoring chemical catalysis whilst micro-scale roughness at the particle size of the microbes favors attachment of more cells. So power P is proportional to the biofilm cell number (i.e. P∝N population cell number) until the biofilm becomes diffusion limited when power will reduce. However, cell quantity is not the only feature. If all cells were growing very slowly, they would not be producing maximum power, if they were killed the power output of the whole MFC would be zero. Power is directly proportional to the metabolic rate ($Q_{met}$), i.e. $P\propto Q_{met}$.

In continuous flow, in steady state, metabolic rate relates to the cells’ growth rate (μ) and in carbon energy limited growth conditions, it is proportional to power (Ledezma et al., 2012), i.e. $Q_{met}\propto \mu \propto P$. The difference between MFC and chemostat is in the analysis of what occurs following mutations in a single cell which confers fitness. The chemostat is well known for its ability to fiercely select for any mutation that gives the cell an advantage in growth rate and/or substrate affinity to the principal substrate. The chemostat is fiercely selective and goes through periodic population sweeps of mutated cells. Any mutation in genes for non-essential pathways will ultimately stop synthesis of proteins that are not necessary for the cell to grow. This saves the cell energy (in the form of ATP) required to drive protein synthesis of the unnecessary enzymes. Saving the energy required for non-essential pathways ensures more energy can go into growth. If a single mutation occurred to one of the cells in the monolayer, all its daughter cells may inherit the genetic change, but the culture as a whole would not be enriched any since the offspring are rapidly washed away. The mutant does not increase its proportion to the whole. There is no selection pressure from growth rate change because the cells survival is assured by its binding affinity to the matrix anode. Only if this is disrupted could you exchange old cells by new cells. Hence the species within the biofilm system itself maintain great stability compared to a chemostat. Mixed culture is possible where many species are syntrophic (i.e. “obligately mutualistic metabolism”) where heterotrophic fermenters utilising a potentially wide range of substrates produce short chain fatty acids as their reduced product. The anaerobic respiring anodophiles alone cannot utilise a wide range of substrates, so rely on the fermenting species. It is unknown how following inoculation the mixed cultures (e.g. *E. coli* and *Shewanella*) develop. If the *Shewanella* was inoculated first and the *E. coli* only following the growth of a saturating monolayer, how would *E. coli* colonise? Do the cells bind to each other, or only onto the electrodes? Does the *E. coli* get desquamated at the same rate as the *Shewanella* species? What happens if *E. coli* is inoculated first and allowed to form a biofilm and then challenged by the introduction of the *Shewanella*? These and many other questions require further research before answers will be found. Even a binary microbe system on a wide range of potential substrates is of little complexity when compared to the complexity that could arise when a very diverse community of 100s of different species are being used. Can diverse mixed culture biofilms remain relatively thin and constant over time? This competition between species is studied using individual-based model of microbial communities and other modelling approaches. If the MFC power output function remains constant and shows steady state growth state, does this imply that there is also an ecological steady state or do the populations of different species periodically shift but still give the same electrical performance? There are many examples of different species that can substitute for each other if one should compete more successfully and take on the burden of fermenting at a fast enough rate in order to keep the *Shewanella* supplied with acetate or lactate to maintain a high growth rate of both species. Another MFC study suggested that electrogenic microorganisms have a higher growth rate than non-electrogenic microorganisms (acetogenic) and they prevail in the biological culture when sludge age is decreased (flow rate increased) (Penteado et al., 2016). As pointed out by Penteado et al. (2016), the lower the sludge age, the faster should be the growth rate of microorganisms remaining in the biological reactor to avoid their wash out.

### 4.11 Synchrony

The concept of synchronous and asynchronous growth is of some interest. Samples of cells taken within a minute of being shed have clearly arrived at the same point in the growth cycle, the point of ejection from the biofilm. This means that the whole sample collected over a short time interval represents a set or group of cells that are all in synchrony with each other. The mother layer is assumed to be asynchronous. The advantage of having synchrony in a sample is that the steady state nature of the system can be probed or tested. If the distribution of growth rates is tightly bound around a mean generation time (e.g. 1.0 h^−1^) the offspring will double their number every mean generation time with a step like plot (time versus biomass) observed, whether measured by cytometer, spectrophotometer, viable count or microscopic enumeration. Taking samples at different times during the day can show repeat demonstration of the degree of synchrony that exists at all timepoints. For the same conditions the plots of data should show the same type and magnitude of interdivision with a time axis showing biomass increase, but in a stepwise (staircase) shape over 2 or 3 generations, reflecting their synchrony. However, the synchronous state decays or weakens (the staircase graph loses sharpness of the edges), possibly up to 5 or 6 generations when growth becomes asynchronous again.

Synchrony can be assessed by comparing the frequency function of interdivision time, f(τ), computed from the equation of Harvey (1972) and/or other synchronous culture equations (Plank and Harvey, 1979) for the generation time distribution of individual organisms. A set of tests every few hours showing the same degree of synchronicity would establish that the MFC biofilm remains asynchronous since the sample represents a 1-min sample of detached progeny which in an hour would represent 1/60th of the total active biofilm. Moreover, if the biofilm itself was growing in synchrony, then the sequential samples would not be identical with each other.

## 5 Outlook

Although at first sight it might seem that MFCs can be described (or explained) using classical ideas from electrode chemistry alone, it soon becomes apparent that for many species of anodophiles (those that directly conduct electrons onto the obliging anode) it is the response of the microorganism to their physicochemical environment rather than the reactions occurring at the electrode surface that dictates the biotransformation properties of the whole system. The oxygen reduction reactions (ORR) occurring at the cathode is itself driven by the supply rate of electrons and protons generated by the bacteria at the anode. Without microbes or with inactive and/or killed microbes *in situ* there is zero power output. So, our theory starts with the living cells that drive the MFC's and explain how they might do this over long-term periods as a continuous steady state biofilm, and show how all other features (e.g. cathodic performance, PEM, internal resistance) are a consequence of the microbial growth and reducing activity driving the system; giving the opportunity of the system control. Variations in the dilution rate permit the study of the metabolic strategies pursued by both mono and mixed (highly divergent culture) or the study of small defined microcosms of mixed isolated species with known properties. Most mesophilic heterotrophs can attain growth rates of about 1 h^−1^. Many of the chemostat models and equations can be used for thin film biofilms but not when diffusion limiting conditions come into play. The model equations are good for defined species of anodophiles, but modifications may become important for studying (a) mixed binary or tertiary cultures and (b) mixed highly diverse microcosms. Assuming a biofilm remains “thin” its steady state condition can be tested by measuring synchrony of detached cells (quick sample) and observe the subsequent outgrowth of sample wells by adding soluble electron acceptor molecules and then measure the subsequent outgrowth (e.g. OD_540nm_) which goes up in steps. More studies are required focussing on both pure or mixed microcosms to show how stable mixed ecologies can remain over long periods of time. It has been proved by others (Cao et al., 2019) that the electricity generation capacity and the ability to adapt to a complex nutrient environment by pure monocultures are limited with narrow substrate specificity which is considerably less than the systems constructed using miscellaneous mixed consortia. However, pure cultures are useful to clarify the electron transfer and biochemical pathways at the microbiological level and further reduce the complexity of a mixed system. Of particular importance is the knowledge that high power outputs are associated with both higher growth rates, higher rates of metabolism and synthesis of new biomass (cell progeny). It is anabolism (the building of new biomass), that recycles carbon and minerals as new building blocks ready for making into new cells. This requires higher rates of nutrient uptake and higher rates of recycling (i.e. utilisation of wastes). These processes do not work in opposition. The higher the electrical energy output the faster the growth rate of the cells (until μ_max_ is achieved) and faster growth rate implies faster recycling (turnover) of all important elements. Optimisation of the microbial growth rates directly influences power output as well as other functionalities directly correlated with MFC performance as MFCs show promising bioremediation potential for the removal or recovery of various inorganic and organic pollutants from wastewater (He et al., 2015), which should be the subject of future studies. Furthermore, it can be expanded into multi-modular scaled-up systems where the control of the multi-MFC stacks and cascades could be greatly improved by using machine learning (ML) tools and AI to manage the peripherals including feedstock supply, in order to maintain stable, optimum levels of power, through constant monitoring of voltage and power profiles.

## 6 Conclusions

By using an appropriately designed MFC, the experimenter can use it as a tool to gain knowledge by its comparison to a chemostat. The methods for calculating growth rate may not apply to thick diffusion limiting conditions, but the model would be useful for thin biofilms, wherever these occur. One focus of this work was to explain the properties of small-scale perfusion anodes to reach steady state conditions at high growth rates with maximum power. Under certain conditions (C/E-limiting) the operator can change the growth rate by changing the flow of medium through the system; akin to a chemostat.

## Credit author statement

John Greenman: Conceptualization, Data curation, Formal analysis, Investigation, Methodology, Visualization, Supervision, Writing – original draft, Writing – review & editing, Funding acquisition. Buddhi Arjuna Mendis: Investigation, Data curation, Formal analysis, Methodology, Writing – original draft, Writing – review & editing. Iwona Gajda: Visualization, Writing – review & editing. Ioannis Ieropoulos: Conceptualization, Project administration, Resources, Supervision, Writing – review & editing. Funding acquisition.

## Declaration of competing interest

The authors declare that they have no known competing financial interests or personal relationships that could have appeared to influence the work reported in this paper.
